# Emergence of Anthrax Edema Toxin as a Master Manipulator of Macrophage and B Cell Functions 

**DOI:** 10.3390/toxins2071881

**Published:** 2010-07-19

**Authors:** Bryan T. Gnade, Scott T. Moen, Ashok K. Chopra, Johnny W. Peterson, Linsey A. Yeager

**Affiliations:** 1 Department of Microbiology & Immunology, The University of Texas Medical Branch, Galveston, TX 77555, USA; Email: btgnade@utmb.edu (B.T.G.); stmoen@utmb.edu (S.T.M.); 2 Center for Biodefense and Emerging Infectious Diseases and Sealy Center for Vaccine Development, The University of Texas Medical Branch, Galveston, TX 77555, USA; Email: achopra@utmb.edu (A.K.C.); jpeterso@utmb.edu (J.W.P.)

**Keywords:** anthrax, edema toxin, macrophage, B cell, cAMP signaling

## Abstract

Anthrax edema toxin (ET), a powerful adenylyl cyclase, is an important virulence factor of *Bacillus anthracis*. Until recently, only a modest amount of research was performed to understand the role this toxin plays in the organism’s immune evasion strategy. A new wave of studies have begun to elucidate the effects this toxin has on a variety of host cells. While efforts have been made to illuminate the effect ET has on cells of the adaptive immune system, such as T cells, the greatest focus has been on cells of the innate immune system, particularly the macrophage. Here we discuss the immunoevasive activities that ET exerts on macrophages, as well as new research on the effects of this toxin on B cells.

## 1. Introduction

Anthrax is an asynchronous, toxin-mediated disease caused by a gram-positive bacterium, *Bacillus anthracis*. This disease has been studied since the early nineteenth century but received much attention after Robert Koch formulated his famous postulates to prove that *B. anthracis* is the causative agent of anthrax. *B. anthracis* relies on an anti-phagocytic capsule and production of two toxins for its immune evasion abilities. Anthrax toxin is a tripartite A-B type toxin consisting of the receptor-binding subunit, protective antigen (PA), and the catalytic subunits, lethal factor (LF) and edema factor (EF). PA binds two cellular receptors that are found abundantly on various host cell types, capillary morphogenesis gene 2 (CMG2 or ANTX2) [1] and tumor endothelial marker 8 (TEM8) [2]. PA is undoubtedly essential for virulence of toxigenic, noncapsulated strains of *B. anthracis*, such as the attenuated Sterne strain, as PA-deficient strains were non-lethal in mice [3,4]. On the other hand, lethal infection with fully virulent encapsulated strains, such as Ames, was not dependent on the presence of PA or other toxin components [5]. Further, Ames-derived mutants that were non-toxigenic remained highly virulent in mice [6]. 

There are three vastly different forms of the disease: cutaneous, gastrointestinal (GI), and inhalational. Cutaneous anthrax, with an estimated 2000 cases reported annually world-wide [7], is naturally acquired when spores breach the epithelial barrier through abrasions in the skin and often remains localized. Neutrophils have been reported to surround the necrotic, bacteria containing tissues [8]. GI anthrax is characterized by two forms of infection (oropharyngeal and lower GI), intestinal abscesses, necrosis, and hemorrhage [9]. Epithelial barrier breakdown is reported in some cases [9], but infections also occurred in the absence of mucosal damage [10]. Peyer’s patches in a mouse model of GI anthrax are the primary site of bacterial growth [10]. The early phase of inhalational anthrax pathogenesis is characterized by alveolar macrophages and dendritic cells (DCs) rapidly engulfing spores that are inhaled deeply within the lungs. While inside the host cells, spores germinate into replicating bacilli. Germination is an astonishingly quick event:evidence has shown that 85% of spores germinate inside murine macrophages within 15 minutes [11]. Although macrophages were once presumed to be the primary mode of transport of spores/bacilli to the mediastinal lymph nodes, a recent publication has provided convincing evidence that DCs are the true “Trojan Horse” of the organism [12]. 

The second pathogenic phase involves extensive toxin production by the replicating vegetative cells. The toxins elicit major pathological consequences; because they can act both while bacilli are enclosed inside the host cells and after the organisms have escaped. Lethal toxin (LT; comprised of LF and PA) is a zinc-dependent metalloprotease that cleaves mitogen-activated protein kinase kinases [13]. LT is cytotoxic to endothelial cells [14], macrophages [15,16], DCs [17] and mononuclear cells [18]. Not clear is the exact mechanism with which the host cell is lysed before the organism is destroyed by the cell’s sporicidal and bactericidal mechanisms. After multiplying in the mediastinal lymph nodes, they disseminate into the host’s bloodstream. *B. anthracis* bacteremia then ensues, with the consequent production of large quantities of the toxins [19]. 

Edema toxin (ET; consisting of EF and PA) is the other toxin produced by *B. anthracis*. Edema factor (EF) is a calcium- and calmodulin-dependent adenylyl cyclase [20] that converts ATP to cAMP with 1000-fold more activity than host adenylyl cyclases [21,22,23]. It belongs to the same class of adenylate cyclases as Exo Y of *Pseudomonas aeruginosa*, adenylate cyclase of *Yersinia pestis*, and adenylyl cyclase toxin of *Bordetella pertussis* [24]. The intrinsic adenylyl cyclase activity of ET upsets the delicate physiological equilibrium inside a wide variety of host cells. For years it was speculated that this would lead to a suppressed immune response, but only recently has this prospect started to be explored [25,26,27,28,29,30,31,32,33,34].

During the early years of research in the field, the role of ET in the pathogenesis of anthrax was generally disregarded. Instead, research efforts were heavily concentrated on the enzymatic activity of LT. The cytotoxic nature and well-described immunosuppressive activities of LT made it the most attractive molecule for further studies. However, the recent culmination of studies suggests that ET plays more than a minor role in the disease process. The interaction of LT with an assortment of host cell types of the innate and adaptive immune system has been thoroughly reviewed in other publications [35,36]. Here, we review findings regarding ET interaction with a key player in the host innate immune defense, the macrophage. We also present new data illustrating the effects of ET on B cell functions that are crucial to the development of an adaptive immune response in the host. 

## 2. Effects of ET on Essential Macrophage Functions

The macrophage is a critical component of the innate immune system. These cells possess an extensive arsenal of weapons designed to eliminate microbial invaders, such as efficient phagocytic capabilities, chemotaxis, activation of cationic proteins and production of defensins and reactive oxygen species. In the context of anthrax infection, macrophages seem to afford protection to the host, as mice that have been depleted of macrophages become more susceptible to the disease [37,38]. This resistance to infection can be further enhanced when mice are reconstituted with macrophages before challenge [37,38]. Thus, it is not surprising that *B. anthracis* has developed methods of suppressing and avoiding certain macrophage functions. It has been shown that macrophages are specifically targeted by LT and ET, because toxin receptor-negative macrophages were able to limit anthrax pathogenesis in mice infected with virulent *B. anthracis* Ames spores [39]. 

### 2.1. Global Gene Changes Induced by ET

In 2006, microarray analysis of ET-treated murine macrophages was among the first evidence of the powerful and wide-sweeping activity belonging to this toxin. Results showed that the expression of a significant number of genes responsible for key cellular functions was modulated after as little as three hours of toxin exposure time [40]. This included genes belonging to categories such as immune response/inflammation, cell signaling, and transcription regulation. As the level of cAMP rose, so did the number of affected genes. After six hours, the number of altered genes in each category had at least doubled. Further, genes involved in apoptosis, adhesion, migration, cytoskeletal structure and metabolism were altered [40]. 

ET-treatment induced activation of the transcription factors AP-1 and C/EBP-β in murine macrophages [40]. Both are major regulators of apoptosis and stress-associated immune responses, therefore, ET-induced activation of these components likely play major roles in affecting macrophage function. Additionally, anthrax toxin receptor 2 (ANTX2) was upregulated at three hours and remained elevated at six hours in macrophages treated with ET [40]. This may represent an evasive strategy unique to this toxin because an increase in ANTX2 transcription may potentially cause macrophages to become more susceptible to binding of PA, thus facilitating entry of both anthrax toxins. 

### 2.2. Intracellular Signaling of ET

The majority of early ET signaling studies involved introducing purified toxin from an extracellular location, mimicking what likely happens during later stages of infection, *i.e.*, when bacilli are replicating extracellularly and secreting large quantities of ET. However, several studies have investigated the effects of intracellular toxin production on macrophages. Banks *et al.* demonstrated in 2005 that toxins produced by germinating spores bind to ANTX2 within the macrophage phagolysosome and kill them from within, but they did not delineate which of the two toxins was responsible for the lethality of the macrophage [41]. Kim *et al.* have provided recent evidence that EF produced from *B. anthracis* bacilli located within murine bone marrow-derived macrophages (BMDM) have the ability to modify cellular activity, such as activating protein kinase A (PKA), inducing CREB phosphorylation, and transcription of a downstream target gene [33].

Data published by Puhar *et al.* revealed a two-phase signaling process by ET. In Jurkat cells, the first intoxication phase is characterized by PKA-dependent signaling, which results in CREB phosphorylation and gene transcription [31]. However, after a prolonged incubation, there is inhibition of subsequent phosphorylation of CREB by incoming stimuli. This is likely another example of the cell’s negative feedback mechanism to control the unrestrained synthesis of ET-induced cAMP. Because CREB phosphorylation is a key step in T cell activation, ET intoxication may prevent an efficient T cell response to invading *B. anthracis* bacilli, thus possibly representing another strategy for immune evasion by the organism. ET has been shown to phosphorylate CREB in multiple other cell types [33,34,42], therefore, the authors proposed that it was possible that the two-phase signaling process described for T cells might also apply to macrophages. Further investigation is needed to clarify this scenario.

In 1998, exchange protein activated by cAMP (Epac) was identified as a member of the cAMP signaling cascade that acts independently of PKA [43,44]. Until this point, the effects of cAMP were thought to be solely transduced by PKA. Epac belongs to a family of guanine exchange factors that directly activate the small GTPase Rap1 and participates in versatile pathways. In macrophages, ET-generated cAMP signals through the PKA and Epac pathways in order to inhibit phagocytosis and actin cytoskeleton remodeling [45]. By signaling via both pathways, ET could elicit a more profound disruption of normal cellular signaling than if only one pathway was perturbed, and it could become more difficult for the host macrophage to regain control of cAMP synthesis. It appears that certain aspects of ET-related intracellular signaling are cell type specific. Human endothelial cells treated with ET underwent cytoskeletal changes and exhibited impaired chemotaxis, but these effects were due to activation of the Epac pathway alone [26]. Previous microarray analysis identified a substantial downregulation of the Epac-related activator of Rap1, Rap guanine nucleotide exchange factor 5 (RapGEF5), following a six hour ET treatment of murine macrophages [40]. 

### 2.3. ET-Induced cAMP Production and Cytotoxicity Is Cell-Dependent

Although differing culture conditions and concentrations of EF and PA have been used in various studies across the literature, it appears that the intensity and duration of the cAMP response generated by ET varies between cell types. For instance, human lymphocytes are much more potent producers of cAMP in response to ET than human neutrophils [46]. ET-treated murine macrophages (RAW264.7 cells) also responded with robust intracellular cAMP levels over a two hour period [46]. Kumar *et a.l* did not measure cAMP concentrations beyond two and a half hours in these experiments, so it is unknown if cAMP levels peaked in these cell types or continually increased over time [46].

We were the first to evaluate cAMP production by ET-treated human macrophages, using both primary monocyte-derived macrophages and a differentiated immortalized cell line (HL-60). ET significantly increased cAMP levels of both cell types by six hours post-treatment, and the effect was further enhanced over a 24 hour treatment period [45].

Another ET-induced effect that appears to be cell dependent is cytotoxicity. For instance, CHO cells were not killed by the toxin, while RAW 264.7 murine macrophages were susceptible to killing [47]. Further, cAMP levels did not correlate with cell death because some cells died in the presence of low cAMP concentrations, while other cell types remained viable despite high levels of cAMP production [47]. The dying cells did not exhibit any hallmarks of the apoptosis or oncosis pathways of cell death [47], so the mechanism of the ET-induced cytotoxicity remains unknown. Identifying the death pathway should be explored further because it could explain how ET treatment causes extensive tissue lesions in an ET mouse model [48]. The viability of human macrophages, both primary and an immortalized cell line, was not affected by ET exposure [45]. The effect of ET on the viability of other immune cells has not yet been reported in the literature. 

### 2.4. ET Alters the Motility and Phagocytic Activity of Macrophages

It appears that the effect of ET on macrophage migration depends on the source of the cells. This is not surprising since the role of the toxins appears to differ between the murine and human models of disease. Kim *et al.* observed that ET treatment of murine BMDM’s significantly increased spontaneous macrophage migration [33]. This corresponded with a redistribution of actin to the cell margin and the formation of filopodia. The use of cAMP analogs with specificity to either the PKA or EPAC pathways revealed that this enhanced migration was linked exclusively to PKA signaling [33]. 

Alternatively, ET exposure caused reduced chemokine-directed migration of human macrophages [32]. This was accompanied by reduced Erk1/2 activation, suggesting that the toxin impaired immune cell homing by interfering with CXC and CC chemokine receptor signaling. The actin cytoskeletons of the immobilized macrophages were not examined in that particular study. However, Yeager *et al.* [45] used immunofluorescence techniques to visualize and measure changes in the cytoskeleton of macrophages as a consequence of ET treatment. We showed that ET caused these cells to appear immobilized and remain rounded, with reduced filopodia, even in the presence of fluorescently-labeled phagocytic particles. We also noted reduced levels of F-actin content in ET-treated cells compared to that of the untreated cells [45]. Similar to Rossi Paccani *et al.* [32], these cells exhibited reduced migration towards MIP-1α (data not shown). 

We were the first to demonstrate the ability of ET to significantly decrease the phagocytic activity of human macrophages [45]. Primary human monocyte-derived macrophages treated with ET were not capable of phagocytosing virulent Ames spores to the same extent as untreated cells. We attributed this to the concurrent observation that the actin cytoskeleton of toxin-treated cells appeared frozen and unable to undergo dynamic remodeling when introduced to phagocytic particles. Actin remodeling is a highly dynamic process and is required in order for macrophages to carry out its normal functions. Thus, the ability of ET to paralyze such an important aspect of this cell type is a dangerous immunoevasive mechanism held by *B. anthracis*. The observed defects in actin rearrangement can be possibly explained by the striking downregulation of multiple genes related to the cytoskeleton [40]. These genes include and code for PDZ and LIM domain protein 2 (PDLIM2), formin-binding protein-1 like (FNBP1L), and switch-associated protein 70 (SWAP-70). 

The ability of ET to reduce the capacity of macrophages to phagocytose spores/bacilli is likely important during the later stages of infection, particularly when large amounts of ET are being secreted by rapidly multiplying extracellular vegetative cells. At this time, ET-induced suppression of macrophage phagocytosis would be of tremendous advantage to the survival of the bacteria and any remaining viable dormant spores. ET also impairs the uptake of Sterne spores by human neutrophils [49]. The culmination of ET crippling two types of the host's professional phagocytes may explain how quickly the organism begins to multiply systemically, suddenly resulting in fulminant anthrax disease. 

### 2.5. Macrophage Cytokine Modulation by ET

Very limited information regarding cytokine production profiles by macrophages treated with purified ET has been reported in the literature thus far. Shen *et al.* and Comer *et al.* showed that ET inhibited TNF-α production by murine BMDMs and RAW 264.7 cells, respectively, as a result of ET exposure, even in the presence of LPS [50,40]. ET also increased IL-6 production in BMDMs [50]. It is likely that this toxin is able to modulate human macrophage cytokine levels because augmented cAMP levels have been shown to influence the production of IL-1, IL-6 and TNF-α in human monocytes [51,52]. Further, cytokine analysis of culture supernatants and mRNA quantitation have shown that human monocytes respond to ET with enhanced secretion of IL-6 [53]. These cells also resulted in reduced production of TNF-α following dual treatments with ET and LPS [53]. Due to the complex interactions between these and other cytokines during infection, it is predicted that this can trigger serious consequences for the host response to anthrax infection. For instance, TNF-α enhances the microbicidal activity of macrophages [54,55], thus hindering its production may allow for increased survival of phagocytosed spores and/or bacilli. 

### 2.6. Synergistic Effects of LT and ET

During an actual anthrax infection, it is expected that both ET and LT are secreted simultaneously. Although a recent publication measured the concentrations of these toxins in the blood of infected rabbits [19], the local concentration of LF, PA, and EF at the site of germinating and replicating organisms remains unknown. A vast number of studies have examined the interaction of each individual anthrax toxin with diverse cell types, but a more accurate portrayal of true physiological consequences must include both types of toxins in the culture conditions. It is speculated that both toxins act synergistically *in vivo* to disable the innate and adaptive immune responses of the host and the evidence reported thus far supports that notion [56]. *In vitro* studies with DCs infected with *B. anthracis* mutants expressing genes encoding LT only, ET only, or both toxins demonstrated a cooperative effect in suppressing cytokine production [29]. The combined mixture of both toxins also collectively inhibited T cell activation [28]. However, the combined effects of LT and ET on macrophage functions have not been reported thus far.

## 3. B Cells: An Unknown Target of ET

A variety of B cell functions are important for the host adaptive immune response to be fully effective. For instance, B cell migration is vital for B cell and T cell interactions. B cell activation is an important step in the adaptive immune response and a number of different cytokines and chemokines are produced by B cells contributing to both the innate and adaptive immune response. Clonal expansion is another B cell function vital to an effective B cell response to an antigen. 

Although still sparse in comparison to what is known about LT, the studies investigating the effects of ET on macrophages and other innate immune cells far surpass the published data describing the interaction between ET and B cells. The effects of ET have been shown to be considerable on a related cellular component of the adaptive immune system, T lymphocytes. For instance, although ET does not appear to affect T cell viability [28], it does impair its activation (inhibition of CD25 and CD69) [28], proliferation [28], cytokine production [27] and chemotaxis [32]. 

In general, the anthrax toxins’ effects on B lymphocytes remains one of the least examined potential mechanisms of immune evasion that *B. anthracis* harbors. Fang *et al.* demonstrated that LT was not lethal for B lymphocytes but did cleave MEK-1 and 2 in PAM- and LPS-stimulated B lymphocytes [57]. Phosphorylation of ERK, JNK and p38 in PAM-stimulated B lymphocytes was impaired by LT [57]. IgM production and proliferation of stimulated B lymphocytes was also significantly impaired in LT-treated B lymphocytes [57]. This study demonstrated that B lymphocytes were indeed targets of LT, but the effects of ET on B lymphocytes were not examined. Consequently, we examined and showed for the first time that ET did indeed impair key B cell functions. 

### 3.1. B Cells Are Susceptible to ET Activity

**Figure 1:**
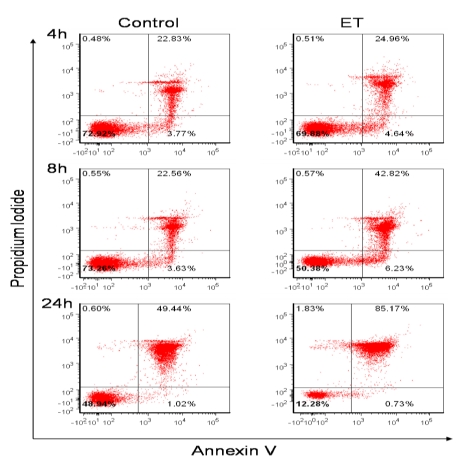
ET effects on B lymphocyte viability. Naïve B lymphocytes isolated from female BALB/c mice spleens using a Miltenyi Biotec B cell isolation kit were resuspended at 1 × 10^6^/mL in RPMI 1640 with 10% FBS, 1 mM sodium pyruvate, 50 μM 2-ME, 100 μg/mL streptomycin and 100 U/mL penicillin. B cells were treated with ET (2.5 μg/mL PA and 1.0 μg/mL EF) for 4, 8, and 24 h. Control cells were untreated. Viability was assessed by flow cytometry after Annexin V (eBioscience) and PI (Sigma) staining. The data are from one experiment and are representative of at least three independent experiments. The standard deviations at the 24 h time point for three individual replicates of one experiment are as follows: Ann^−^/PI^−^ 47.3 ± 0.95 control and 10.83 ± 1.38 ET; Ann^+^/PI^−^ 1.01 ± 0.4 control and 0.63 ± 0.1 ET; Ann^+^/PI^+^ 48.6 ± 0.93 control and 89.9 ± 1.32 ET; Ann^−^/PI^+ ^0.6 ± 0.1 control and 1.95 ± 0.51 ET.

Because B cell viability following ET exposure has not been reported previously, this was the first parameter we examined. It has previously been reported that increases in cAMP induce B cell apoptosis [58], thus it was hypothesized that apoptosis would be the mechanism of toxin-mediated cell death. Therefore in our studies, murine B lymphocytes were treated with ET and potential cell death was measured by Annexin V and Propidium Iodide staining. As Figure 1 shows, there was a noticeable decrease in the percentage of healthy untreated control cells (50.38% AnnV^−^/PI^−^) in the ET group as early as 8 h. This correlated with an increase in the percentage of AnnV^+^/PI^−^ cells in the ET group at 8 h (6.23%), suggesting that some cells were undergoing early apoptosis. There was also an increase in the percentage of AnnV^+^/PI^+^ cells (42.82%) as a result of ET-treatment at this time point, which is indicative of late apoptosis and/or the cells that were already dead. By 24 h, the decrease in toxin-treated B cell viability was even more dramatic (Figure 1; only 12.28% AnnV^−^/PI^−^ ET-treated cells). However, only 0.73% of the ET-treated population was in the early apoptotic stage at 24 h, while the AnnV^+^/PI^+^ (late apoptotic or already dead cells) population increased to 85.17%. There was also a small increase in the percentage of AnnV^−^/PI^+^ cells at 24 h (1.83%), which suggested that a small portion of cells were possibly undergoing necrosis. Importantly, because this staining method does not clearly discriminate between cells in late apoptosis or necrosis, additional experiments involving multiple other time points are presently being conducted to further identify and characterize the mechanism of ET-induced B cell death.

**Figure 2:**
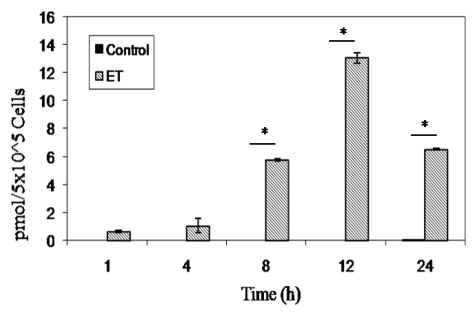
cAMP production in ET-treated murine naïve B lymphocytes. 5 × 10^5^ naïve B lymphocytes in 200 μL were plated in each well of a 96 well tissue culture plate and treated with ET (2.5 μg/mL PA and 1.0 μg/mL EF) and intracellular cAMP was measured using a direct cAMP Enzyme Immunoassay kit according to manufacturer’s instructions (Assay Designs). Each bar represents the mean ± standard error of triplicate values. The data are from one experiment and are representative of three independent experiments. Asterisks denote a statistically significant difference between untreated and ET-treated cells (p < 0.05, ANOVA followed by the Student’s t-test).

Fang *et al.* demonstrated that PA binds to B cells allowing LF to enter [57]. To determine if EF was able to bind/enter B lymphocytes through interaction with PA, we exposed naive murine B lymphocytes to ET for 1, 4, 8, 12, and 24 h. As expected, ET treatment resulted in significant increases in cAMP over controls at 8 h and beyond, peaking at 12 h after exposure (Figure 2), which suggested EF binding and possible entry of this toxin in B cells.

### 3.2. Anthrax ET Inhibits B Cell Migration

**Figure 3:**
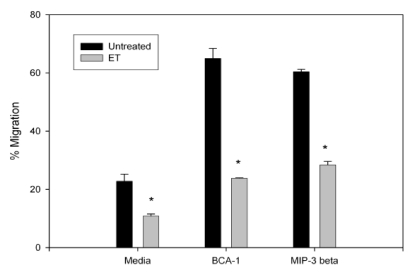
Migration of ET treated naïve murine B lymphocytes. An aliquot (5 × 10^5^) of naïve B lymphocytes were incubated with ET (2.5 μg/mL PA and 1.0 μg/mL EF) for 4 h and added to the upper wells of 5-µm pore transwell migration chambers. The lower wells contained media alone or media with BCA-1 (1 μg/mL) and MIP-3β (1 μg/mL) (R&D Systems). Cells were incubated for 3 h and migrated cells were stained with Trypan Blue and quantitated. Values are the percent of cells added to the upper wells that migrated to the lower wells. The bars represent the mean ± standard deviation of two values. The data are from one experiment and representative of three independent experiments. Asterisks denote a statistically significant difference between untreated and ET-treated cells (p < 0.05, ANOVA followed by the Student’s t-test).

**Figure 4:**
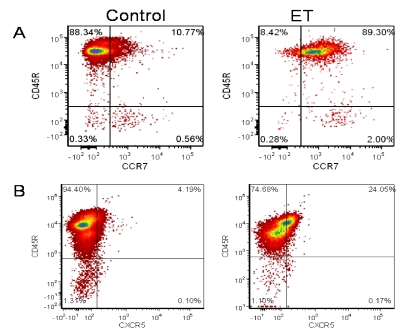
CCR7 and CXCR5 expression on toxin-treated naïve B lymphocytes. 1 × 10^6^/mL naïve B lymphocytes, isolated from female BALB/c mice spleens, were treated with ET (2.5 μg/mL PA and 1.0 μg/mL EF) and incubated for 24 h at 37 °C and 5% CO_2_. Cells were then stained with fluorochrome labeled anti-CCR7 (eBioscience) and anti-CXCR5 (BD Bioscience). CCR7 (**a**) or CXCR5 (**b**) expression was then measured using a FACSCanto. The numbers indicate % positive cells. The data are from one experiment and representative of at least three independent experiments.

Migration of B lymphocytes is vital for an effective humoral response following bacterial challenge. In the lymphoid organs, B lymphocytes localize to the B cell follicles due to CXCR5 surface expression and local CXCL13 expression [59]. CCR7-mediated migration is required for B cell accumulation in the lymph nodes and migration to the B cell - T cell boundary [60,61]. Therefore, to determine the effects of ET on B cell migration, B lymphocytes were collected and treated with the ET for 4 h. ET decreased both basal migration and chemokine-directed migration (BCA-1 and MIP-3β chemoattractants; Figure 3). To determine whether this impaired migration was due to a change in chemokine receptor expression, treated cells were stained for CCR7 and CXCR5, the receptors for MIP-3β and BCA-1, respectively. Despite the ET-induced decrease in migration towards both chemokines, ET-treated cells had an increase in CCR7 expression (Figure 4A) but no effect on CXCR5 expression (Figure 4B). Previous reports of migration impairment in the HL-60 cell line showed a decrease in F-actin content in ET-treated cells [45], and it is a possible explanation for the migration impairment observed here.

### 3.3. ET-Induced Modulation of B Cell Cytokine Production

**Figure 5:**
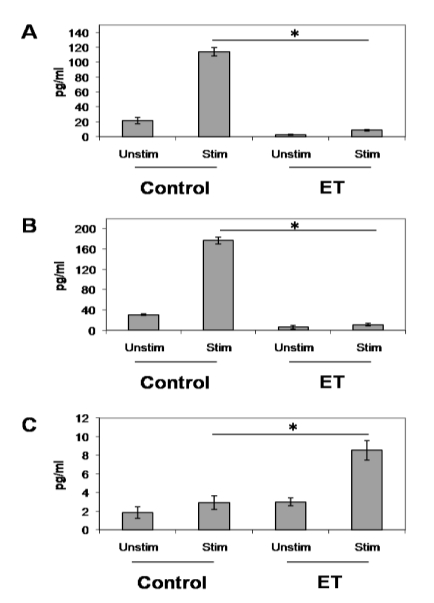
ET’s effect on cytokine and chemokine production in stimulated B lymphocytes. 1 × 10^6^/mL naïve B lymphocytes from Balb/c mice were treated with ET (2.5 μg/mL PA and 1.0 μg/mL EF) for 4 h at 37 °C and 5% CO_2_. Cells were then stimulated with 5 µg/mL anti-CD40 antibody, 5 ng/mL IL-4 and 5 µg/mL anti-IgM antibody (eBioscience) for 48 h. After 48 h, supernatants were collected and levels of MIP-1α (**a**), MIP-1β (**b**) and IL-6 (**c**) were measured using a multi-plex cytokine array kit (Millipore). The data are expressed as means ± standard deviation of triplicate values and are representative of two independent experiments. Asterisks denote a statistically significant difference between untreated and ET-treated cells (p < 0.05 by ANOVA and Student’s t-test). Unstim—unstimulated; Stim—stimulated B cells.

Antigen-stimulated B lymphocytes release the important T cell chemoattractants, MIP-1α and MIP-1β [62]. These chemoattractants play an important role in facilitating the B/T interactions that interrupt the B cell receptor-induced cell death program. Here, we showed that MIP-1α and MIP-1β production by stimulated B lymphocytes was impaired by ET at 48 h (Figure 5A and B). This could potentially impair the migration of T cells to antigen-stimulated B lymphocytes resulting in the absence of a CD40/CD40L cell interaction, and subsequent programmed cell death in these B lymphocytes without producing memory B lymphocytes and high-affinity antibodies. Alternatively, IL-6 production became significantly increased following ET exposure (Figure 5C). This is not surprising because cAMP has been shown to increase IL-6 gene expression and enhance secretion of biologically active IL-6 [63]. Therefore, we believe that this increase in IL-6 production is a direct result of the increase in intracellular cAMP caused by the adenylyl cyclase activity of ET. Taken together, these data offer further evidence that ET upsets the development of an effective immune response, likely allowing the bacteria a foothold to cause serious infections. 

### 3.4. ET Alters B Cell Surface Marker Expression

**Figure 6:**
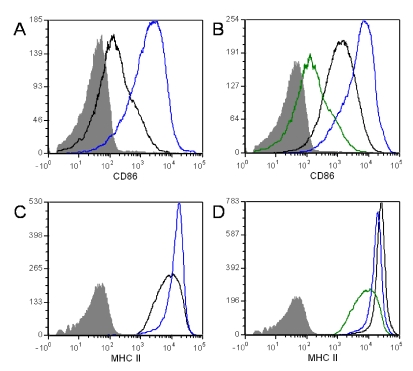
Expression of surface markerson toxin-treated naïve B lymphocytes. Naïve B lymphocytes from Balb/c mice were exposed to ET (2.5 μg/mL PA and 1.0 μg/mL EF)for 4 h at 37 °C and 5% CO_2_. After 4 h, cells were left unstimulated (**a**)and (**c**) or stimulated with 5 μg/mL anti-CD40 antibody and 5 ng/mL IL-4 (**b**) and (**d**) for 24 h. Cells were then stained with fluorochrome labeled anti-CD86 and anti-MHCII (eBioscience). CD86 (**a**) and (**b**) and MHCII surface expression (**c**) and (**d**) were measured using a FACSCanto. The data are from one experiment and are representative of at least three independent experiments. In (**a**) and (**c**), the blue line represents ET treated cells and control cells are represented by the solid black line. In (**b**) and (**d**), unstimulated cells are the green line, ET treated cells are blue and control cells are the solid black line. The shaded area represents the isotype control.

A number of B cell surface markers are up-regulated when B lymphocytes are activated, including CD86 and MHCII. The up-regulation of these markers is important for T cell/B cell interactions and antigen presentation. Here we show an increase in both CD86 and MHCII surface expression in ET-treated naïve B cells (Figure 6A and C). This was an unexpected observation for naïve cells, however, cAMP has been shown to induce CD86 (B7-2) expression in macrophages. Further, B7 expression was shown to be elevated in cAMP-treated B cells [64], possibly explaining these results. 

ET treatment resulted in increased CD86 expression in activated B cells (Figure 6C). This is not surprising because it has been demonstrated previously that cAMP analogs enhance CD40-mediated B cell activation [65]. Alternatively, ET exposure slightly impaired MHCII expression when stimulated by anti-CD40/IL-4 (Figure 6D). This disturbance in MHCII expression could have dramatic effects on the formation of T cell-B cell conjugates. This interaction is needed to induce the appropriate signals, such as cytokines and up-regulation of CD40L, from the T cell in order to achieve B cell proliferation and differentiation into plasma cells and memory B cells.

## 4. Conclusions

Undoubtedly, ET is a powerful weapon possessed by the biothreat agent *B. anthracis*. This became particularly evident when Firoved *et al.* demonstrated *in vivo* that ET elicited death at lower doses and more rapidly than LT in a mouse model [48]. Extensive tissue lesions were observed and death was likely due to multi-organ failure. *In vitro* studies with various host immune cell types performed in recent years have contributed to our overall knowledge of the immunosuppressive and immunodeviation strategies of ET. Because cAMP is a second messenger molecule involved in such a wide variety of processes, it is no surprise that perturbation of its synthesis by ET results in a complex web of downstream events. For instance, immune cell migration appears to be a major target of ET because it is common to multiple cell types [26,32,45]. However, gaps still remain in our understanding of ET activity with regard to the macrophage and B cell. Only by building a thorough comprehension of the interference in signaling and function that occurs within these cell types will we potentially discover new therapeutic strategies for the treatment of anthrax in human patients. 

Additional research is needed to determine the mechanism of ET-mediated B cell death. Determining whether the cytotoxic effect of ET extends to memory B cells and plasma cells is another important avenue, considering the rapid waning of antibody titers in Anthrax Vaccine Adsorbed (AVA) recipients. Previous reports indicate that ET impaired cell signaling by reducing the activation of ERK, MEK, and JNK in murine T cells in a cAMP dependent manner [28]. Whether ET exerts this same effect on B cells should be investigated. Further, does ET inhibit antibody production and class switching? Are these effects wide spread across all mammalian species? We are currently pursuing answers to these questions and others. The use of EF^−^ mutants in an infection model will also help elucidate the impact of this toxin on B cells during *B. anthracis* infection.
